# Digital transformation, green innovation and the Solow productivity paradox

**DOI:** 10.1371/journal.pone.0270928

**Published:** 2022-07-08

**Authors:** Shujun Sun, Lin Guo

**Affiliations:** School of Economics, Zhejiang University, Hangzhou, China; Institute for Advanced Sustainability Studies, GERMANY

## Abstract

To provide evidence at the micro level for cracking the Solow productivity paradox, this paper deeply studies the impact of enterprise digital transformation on green innovation. In terms of theoretical research, three potential mechanisms are excavated for the first time; considering empirical research, a series of strict causal effect identification strategies are carried out. The results show that enterprise digital transformation can significantly promote green innovation, and it passes a series of robustness tests and endogenous tests. According to the theoretical and empirical results, the policy suggestions mainly include five points: helping enterprises to accelerate digital transformation; strengthening the green innovation ability of enterprises; reducing internal and external costs and promoting the professional division of labor; piloting the digital transformation policy; enhancing corporate social responsibility. It provides a reference of experience and a path for other countries to follow in implementing a digital transformation strategy and green sustainable development strategy.

## Introduction

The ecological environment is a topic of common concern all over the world, which poses challenges for the sustainable development of economy and the entire human society. Technological innovation is the fundamental driving force of economic development, which determines the upgrading of economic development from an extensive to an intensive form. The advancement of technological innovation influences the ecological environment and vice versa, managing the ecological environment requires technological innovation. The resulting "green innovation" can help enterprises achieve a balance between economic performance and environmental performance [1–3], and has become an important contributor to achieving a win-win situation regarding economic and social development [4]. At the same time, today’s society has quietly entered the era of digital economy. Digital technologies represented by big data, cloud computing, artificial intelligence, blockchain technology, and the Internet of things deeply enable the real economy, break the old kinetic energy, cultivate and develop new kinetic energy, and release huge digital dividends. The United States, The United Kingdom, Germany, France and other developed countries have successively formulated digital economic development policies(The United States has formulated the ‘Digital Economy Agenda’, the United Kingdom has formulated the ‘Digital Britain’, Germany has formulated the ‘Digital Germany 2015’, and France has formulated the ‘Industrial New France 2.0’.). The rapid development of digital economy, the range of radiation and the degree of influence have reached unprecedented levels. This digital wave is globally the new general trend, which is becoming a key force to restructure global factor resources, reshape the global economic structure and change global competition patterns.

In the new context of the explosive growth of the digital economy and its deep integration with the real economy, the study of the performance of the digital transformation of enterprises has increasingly become a focal issue that has been focused on by academia [5, 6]. This naturally leads to a fundamental question of interest in this paper: Does the digital transformation of enterprises promote green innovation? The answer to this question is not only of great practical importance, but also of great theoretical value, as it provides new evidence for deciphering the long-debated Solow Productivity Paradox (SPP) in the academic community.

Robert Solow [7] stated that “You can see the computer age everywhere but in the productivity statistics”, which aimed to criticize the fact that information digital technology (IDT) does not improve the productivity and innovation level. This is the famous SPP, which has aroused extensive and profound arguments among academics since then [8, 9]. The solution to this paradox has been challenging. The mainstream explanation tends to attribute the explanation to neglecting its positive externalities in the process of statistical calculation of IDT. For example, IDT not only improves production efficiency, but also optimizes product design functions and enhances social services [10–12]. Therefore, it is unreasonable to judge the role of IDT only by productivity indicators. Wen et al. [13] also pointed out that IDT shoulders more social goals, such as improving the ecological environment, which cannot be reflected in statistical indicators. It can be inferred from the existing mainstream theoretical explanations that, although IDT may not simply improve productivity, it is expected to upgrade the compound productivity after incorporating the impact of positive externalities. In this way, the SPP has been effectively cracked. However, this is only a theoretical inference, which needs to provide empirical evidence from real data. With regards to carrying out empirical tests, the key is to find a good composite productivity index. The green innovation mentioned above naturally becomes an ideal composite index because it contains both productivity and ecological environmental elements and has the dual externality characteristics that other traditional innovations do not possess [14]. Therefore, we can effectively identify the causal relationship between IDT and green innovation to test the correctness of the mainstream explanation. Thus far, some academic literature has provided evidence at the macro level, however, there is little evidence at the micro level.

Currently, there are few normative empirical studies on the relationship between digital transformation and green innovation, and these are mainly concentrated at the macro level of industry, such as provinces, cities and countries [15–18]. It is generally agreed that digital transformation promotes green innovation and provides macro-level evidence for solving the SPP, and there is limited empirical evidence at the micro level, with Wei and Sun [19] as one of the few representative literatures. They have empirically tested whether the digital transformation of manufacturing enterprises can promote green process innovation by using the cross-sectional data of 334 manufacturing enterprises in China. The results showed that the digital transformation of manufacturing enterprises significantly improves green process innovation. At the same time, the impact is also regulated by horizontal information sharing, technology modularization and vertical bottom-up learning. Their research focuses on the direction of production management and information processing, which has brought great inspiration to this paper, but three deficiencies remain. First, there are sample selectivity errors, mainly because the data samples come from the sampling questionnaires of a small number of enterprises in the manufacturing industry, and the cross-sectional data cannot reflect the changes in the time trend, resulting in the weak external validity of the conclusions. Second, the enterprise digital measurement is limited to the production process, and the construction process is relatively simple, which cannot accurately reflect the overall digital transformation of the enterprise. Third, the identification of causal effects is neither robust nor rigorous. There is an obvious two-way causal endogeneity between digital transformation and green innovation.

Based on the lack of existing literature, the main work of this paper are as follows: Firstly, this paper deeply discusses the potential mechanism of the impact of enterprise digital transformation on green innovation theoretically and puts forward three hypotheses to be tested. Secondly, the text analysis method is used to better describe the digital transformation of enterprises, and then the whole industry data of listed companies are applied to strictly test the relationship between digital transformation and green innovation; Thirdly, a series of robustness tests and endogenous discussions are carried out to strictly identify the causal effects, and the potential mechanisms are also tested; Fourthly, based on theoretical and empirical analysis, this paper puts forward some policy suggestions to promote enterprise digital transformation and green innovation.

Compared to the existing research, the contribution of this paper can be defined in three main points: First, at the theoretical level, it deeply excavates the three potential mechanisms of enterprise digital transformation and green innovation for the first time, which helps to carefully describe the impact path of digital transformation, deepen the understanding of the impact connotation of digital transformation, and provide a solid theoretical basis and analytical framework for solving the SPP. Our study greatly enriches the existing theoretical research on the impact of digital transformation. Second, at the empirical level, it carries out strict causal effect identification and mechanism test, as well as heterogeneity analysis for the first time, expands the existing empirical research ideas and technical methods, provides empirical evidence at the micro level for solving the SPP, and greatly enriches the existing empirical research on the impact of digital transformation. Third, at the practical level, it provides a basis and theoretical support for decision-makers to formulate relevant policies, in order to accelerate enterprise digital transformation and promote the improvement of green innovation level. Moreover, it provides a reference of experience and a path for other countries to follow in implementing a digital transformation strategy and green sustainable development strategy.

The rest of this paper is arranged as follows: The second part combs the existing relevant literature and puts forward three hypotheses to be tested through mechanism analysis. The third part includes the empirical design, including data source, variable definition, descriptive statistics and measurement model setting. The fourth part contains basic regression analysis and robustness analysis. The fifth part discusses the potential endogeneity of the model. The sixth part is to test the mechanism. The seventh part analyzes the heterogeneity and investigates the differential performance of enterprise digital transformation and green innovation. The final part comprises the conclusion and policy recommendations.

## Literature review and theoretical hypothesis

### Literature review

#### Digital transformation and green innovation

In fact, there is very limited normative literature on the relationship between digital transformation and green innovation, and it is mainly concentrated at the macro level. Li et al. [10] empirically tested the impact intensity and potential impact mechanism of Internet development on green total productivity based on the panel data of Chinese provinces from 2009 to 2017 using a fixed effect model and a threshold regression model. They found that the development of Internet has a significant positive role in promoting green total factor productivity and a nonlinear impact at different levels of the human capital. Nguyen et al. [16] took 13 countries in the G20 group during the period of 15 years as the research object, and empirically studied the impact of information and communication technology on economic growth and carbon emissions. They found that the information and communication technology impede carbon emissions and is positive driving factor for economic growth. Based on the panel data of 277 cities in China for 2011–2018, Li et al. [15] constructed the digital economy index and green economy efficiency index. They reported that the digital economy significantly improves the efficiency of the green economy. Ye and Xu [17] empirically tested the impact of environmental regulation on green technology innovation under the background of digital economy by using China’s provincial panel data for 2011–2018. The results showed that digital economy has a positive impact on green technology innovation and helps to reduce the inflection point of the intensity of environmental regulation. Zhou et al. [18] used the panel data of 28 manufacturing industries in China for 2013–2018 to empirically test the impact of the digital transformation of manufacturing industry on green innovation performance. The results showed that digital technology transformation and digital innovation capability transformation have a significant impact on green R&D performance, green manufacturing performance and green service performance.

Currently, only one study at the micro enterprise level is closely related to this paper: Wei and Sun [19] empirically tested whether the digital transformation of the production process could promote green process innovation by using the questionnaire data of 334 manufacturing enterprises in China. The results showed that the digital transformation of the production process significantly improves green process innovation. At the same time, the impact is also affected by the level of information sharing technology modularization and vertical bottom-up learning. That research brought inspiration to the current paper, but there are still some issues to be addressed: first, the data samples were from a small number of enterprise sampling questionnaires in the manufacturing industry. Considering that there are great differences among industries, for example, the manufacturing industry is more likely to undergo digital transformation, and there is great subjectivity and randomness in the process of filling in the questionnaire, these will lead to serious sample selection errors, and the cross-sectional data cannot reflect the time trend changes affected by digital transformation, resulting in the weak external validity of the conclusions of that article. Second, the calculation of digital transformation is only limited to the stage of enterprise production process, ignoring the digital transformation of other departments of the enterprise, such as the use of ERP system in human resources department, the application of OA system in comprehensive management department, and the decision-making of strategic management department on enterprise digital transformation. Moreover, the construction process is relatively simple and direct, which cannot accurately reflect the overall picture of enterprise digital transformation. Third, the causal effect identification is not strict. There is obvious two-way causal endogeneity between digital transformation and green innovation, but this has not been effectively handled, resulting in biased results. At the same time, there is a lack of robustness test of the conclusions, and the estimation results may be unstable. Hence, this paper aims to improve robustness and expand on the basis of existing research.

#### Digital transformation and other micro effects

Thus far, numerous articles have explored the impact of digital transformation on environmental pollution, and there are great disputes both theoretically and empirically [20]. Salahuddin and Alam [21] used a panel data analysis for OECD countries and found that 11% increase in Internet users increased per capita electricity consumption by 0.026%. Afzal and Gow [22] used the dynamic panel data model to find a positive relationship for 11 countries from 1990–2014. Beier et al. [23] argued that the Industrial Internet of Things enabled more resource-efficient manufacturing, improved recycling processes and predictive maintenance. Predictive studies suggest that applying digital devices and programs can increase energy efficiencies in various sectors. Haseeb et al. [24] found a unidirectional causal link running from ICT towards electricity consumption with a panel data analysis for BRICS countries. However, other literature suggests that this positive relationship does not hold true for all countries. Schulte et al. [25] invested sectors in OECD countries and obtained the result that a 1% increase in ICT capital could reduce energy demand by 0.235%. Khayyat et al. [26] regarded that ICT investments were a substitute for both energy and labor in some industrial sectors in South Korea and Japan. Buhl et al. [27] conducted a living lab study and showed that energy consumption remained roughly the same. To this end, Berkhout and Hertin [28] and Beier et al. [23] put forward a theoretical framework to reconcile the disputes. This includes two effects: one is the direct effect, that is, the digital process improves the energy consumption and the pollution level, and the other is the indirect effect, that is, the digital process improves the production efficiency, enhances the product structure and then reduces the pollution level. Wen et al. [13] composed a detailed review of relevance, which will not be discussed herein.

At present, there are many documents on the impact of digital transformation on innovation. Some of them support the effect of digital transformation on innovation, though some scholars believe that this effect is not significant, or digital transformation even inhibits innovation. Huang et al. [29] prepared a more detailed literature review on this topic, which is beyond the scope of this paper.

Some scholars have also recently focused on other micro effects of digital transformation of enterprises: Wang et al. [30] demonstrated the micro mechanism of informatization affecting enterprise capacity utilization and tested it using survey data from 120 prefecture-level cities in China with a total of 12,400 enterprises counted by the World Bank, and found that increasing investment in informatization at either the enterprise or regional level significantly improved the capacity utilization of enterprises. Huang et al. [31] found that Internet development significantly improved the efficiency of manufacturing enterprises by building a theoretical model of the impact of Internet development on manufacturing efficiency, and the results of the intrinsic mechanism test showed that Internet technology development enhanced productivity by reducing transaction costs, reducing resource mismatch, and promoting innovation. Using micro-enterprise data, Liu et al. [32] empirically tested the impact of enterprise digital transformation on organizational empowerment behavior and its core mechanisms by considering organizational empowerment as an important feature of organizational change, and found that enterprise digital transformation weakened executive power, enhanced grassroots power, and induced downward organizational empowerment.

#### Related research on green innovation

The study of green innovation originated in the 1990s and mainly refers to green technology innovation. The World Intellectual Property Organization (WIPO) defines the broadest range of green innovations to include environmentally relevant pollutant disposal and climate change mitigation-related technologies, and provides a list of all relevant technologies with patent classification numbers of all relevant technologies available for researchers to use. Regarding the analysis of factors affecting green innovation, Hojnik and Ruzzier [33] conducted a systematic review, which, in general, mainly includes command-based regulatory policies, market-based regulatory policies, and firm organizational structures. Among them, command-based regulatory policies involve voluntary emission reduction programs, environmental enforcement, etc. [34]; market-based regulatory policies involve environmental equity trading, carbon emissions trading, sulfur dioxide emissions trading, etc. [35]; firm organizational structures involve corporate stakeholder pressure, environmental quality management system, etc. [36, 37].

### Theoretical hypothesis

#### Improvement of information processing capability

Barbieri et al. [38] assumed that the level of green innovation of enterprises depends on the overall analysis of massive information in production and manufacturing links, and that digital transformation can greatly improve the ability of enterprise information analysis and processing, which will directly enhance the level of green innovation of enterprises. When an enterprise’s digital transformation level is high, it can detect and obtain the detailed and massive information of production links through digital technology equipment such as sensors and the Internet of things. Compared with manual data collection, the former is more efficient and accurate [19]. The obtained data can be analyzed and processed through digital technologies, such as big data, cloud computing and artificial intelligence, which can produce a large amount of high-quality and valuable information on production links [39]. The complex causality behind multi-dimensional parameters can be effectively identified through algorithm analysis, which is conducive to stimulating enterprises to identify innovation opportunities [19], so as to greatly improve the production efficiency of enterprises. At the same time, with the help of digital technology and big data information, enterprises can observe the energy consumption and pollution emission in all links of production in real-time, and at the same time, realize energy conservation and emission reduction through production process reengineering and improving the energy consumption mode. In addition, since it is difficult for enterprises to process and integrate the data information of traditional manufacturing systems for green upgrading, enterprises are reluctant to carry out green innovation [40]. However, the enhancement of information processing capacity can improve the willingness of enterprises to perform green innovation, thus indirectly improving its level. To sum up, the following hypotheses are put forward:

H1: Digital transformation improves the green innovation level of enterprises by improving their information processing ability.

#### Reduce internal and external costs

In the process of green innovation, enterprises will produce a lot of internal and external costs, which seriously hinders their incentive to carry out green innovation. First of all, enterprise innovation requires a large amount of R&D capital [41, 42], with long cycle, high risk and uncertain return, which easily brings a bankruptcy risk [43], greatly increasing the management and control cost of enterprise R&D investment. At the same time, the innovation process requires the cooperation and coordination of multiple departments [5, 38],such as the logistics department purchasing and providing R&D raw materials and equipment in time, and the financial department pulling down the accounts required for R&D, the personnel department recruiting and training the required R&D talents in due course, and the comprehensive management department promptly coordinating the communication between the R&D department and other departments, which will generate a lot of internal communication and coordination costs. Secondly, under the constraints of external environmental regulation and enterprise social responsibility awareness, enterprises must incorporate environmental factors into the innovation process [44, 45], improve production processes, introduce clean production equipment, and increase front-end green investment. At the same time, they also need to purchase pollution treatment equipment and increase back-end green investment. According to the neoclassical economic theory, this will bring additional environmental costs to enterprises, thus increasing production and operating costs. Thirdly, according to the transaction cost theory, enterprises will also face external costs such as research, in-house negotiation and negotiation with the outside world. This paper holds that the digital transformation of enterprises can reduce the internal and external costs of enterprises through the following aspects, and then stimulate green innovation: First, digital technology can accelerate the diffusion of information, help enterprises to understand the information of potential counterparties, and reduce the search cost that enterprises need to pay [46]. Second, digital technology can reduce the negotiation cost required by enterprises in the process of signing contracts [47, 48]; Third, digital technology can enable organization and management through the management information system and the financial information system [32], reduce the coordination cost of all links of enterprise production and improve the efficiency of enterprise management decision-making [49]. To sum up, the following hypothesis is put forward:

H2: Digital transformation improves the level of green innovation by reducing the internal and external costs.

#### Division effect

Yuan et al. [48] proved through theoretical and empirical analyses that enterprise digital transformation can promote the enterprise division of labor. The underlying mechanism is that digital development makes it easier for enterprises to obtain information, and reduces the coordination cost between enterprises and the market. Enterprises will resell some internal businesses with high production costs to external markets with higher production efficiency, so as to promote the professional division of labor of enterprises [47]; meanwhile, the specialized division of labor in enterprises can improve labor productivity and promote technological innovation [50]. In addition, after the specialized division of labor, the enterprise will focus all human, material and financial resources on the most efficient production links to maximize profits under limited investment. During the sample period, the government has introduced a series of environmental regulation policies and the concept of green and high-quality development, which, as mentioned in the second mechanism, will force enterprises to improve their social responsibility. Then, the enterprise will resell some production links with low efficiency, high energy consumption and high pollution to a more efficient and environment-friendly external market, in order to realize the transformation from extensive multi production link mode to intensive, efficient, single production mode with simplified transformation, reducing energy consumption and pollution emission, which in turn and realizes a clean production mode. To sum up, the following hypothesis is put forward:

H3: Digital transformation improves the level of green innovation of enterprises by promoting the division of labor.

In summary, digital transformation can promote green innovation in firms by improving their information processing ability, reducing the internal and external costs and promoting the division of labor. Thus, the SPP can be effectively solved. Next, this paper will conduct a rigorous empirical test.

## Empirical design

### Data sources

The basic data of this paper are mainly from A-share listed companies of the China Stock Market & Accounting Research Database (CSMAR). Considering that the digital economy developed rapidly only after 2010 [48], and the green list of international patent classification was only launched in 2010, the data are limited to the period of 2010–2018. With reference to the mainstream literature practice [48, 51, 52], the raw data were cleaned as follows: (1) exclude companies in financial and insurance industries; (2) exclude Growth Enterprise Market(GEM) listed companies; (3) exclude ST, PT and insolvent companies; (4) exclude missing values in the regression variables; (5) exclude observations that do not meet the general accounting criteria, such as negative total assets, negative subsidy income, etc. Finally, 7625 observations of 1702 companies are obtained. In addition, the patent data of listed companies are from the State Intellectual Property Office (SIPO) and World Intellectual Property Organization (WIPO). The annual report data of listed companies are extracted from cninfo.com, the social responsibility data of listed companies are obtained from hexun.com, and the provincial data are mainly gathered from the China Statistical Yearbook and Report on China’s provincial marketization index.

### Definition of variables

#### Enterprise green innovation

Enterprise green innovation (‘Green’) is the explained variable of this paper. Xu et al., Wang and Zhao [53, 54] believe that green patents have the inherent advantages of being a measure of green technology innovation, hence this paper decides to use the number of green patents to measure enterprise green innovation. Based on the practices of Qi et al., Wang et al., Zhong and Yang [55–57], firstly, we obtained the patent application and patent grant data of listed enterprises from the search page of the State Intellectual Property Office of China (SIPO). Secondly, according to the "International Patent Green Classification List" launched by the World Intellectual Property Office (WIPO) in 2010 (available at https://www.wipo.int/classifications/ipc/en/green_inventory/), there are seven major categories of green patents, including transportation, waste management, energy conservation, alternative energy production, administrative regulatory or design aspects, agriculture or forestry, and nuclear power generation. Thirdly, based on the above classification criteria, we identified and summed up the number of green patents at the firm-year level, and further distinguished between green invention patents and green utility patents.

Here, there are two problems to be solved: first, whether we should use green patent application or green granted patent for measurement. Qi et al. [55] considered that green patent application only reflects the importance enterprises attribute to green technology, not the actual improvement of green technology, and granted green patents better reflect the actual green innovation ability of enterprises. Therefore, this paper uses granted green patents to measure the green innovation of enterprises. The second question is whether to use the proportion of granted green patents or their absolute number to measure green innovation. Popp [58] believed that the proportion of green patents can more effectively eliminate other unobservable factors that promote enterprise innovation than the number of simple patents. Therefore, we measure enterprise green innovation by the ratio of green granted patents to the total number of patent applications in that year. To ensure the robustness of the results of this paper, other measurement methods are also used for testing, as detailed below.

#### Enterprise digital transformation

**Fig 1:**
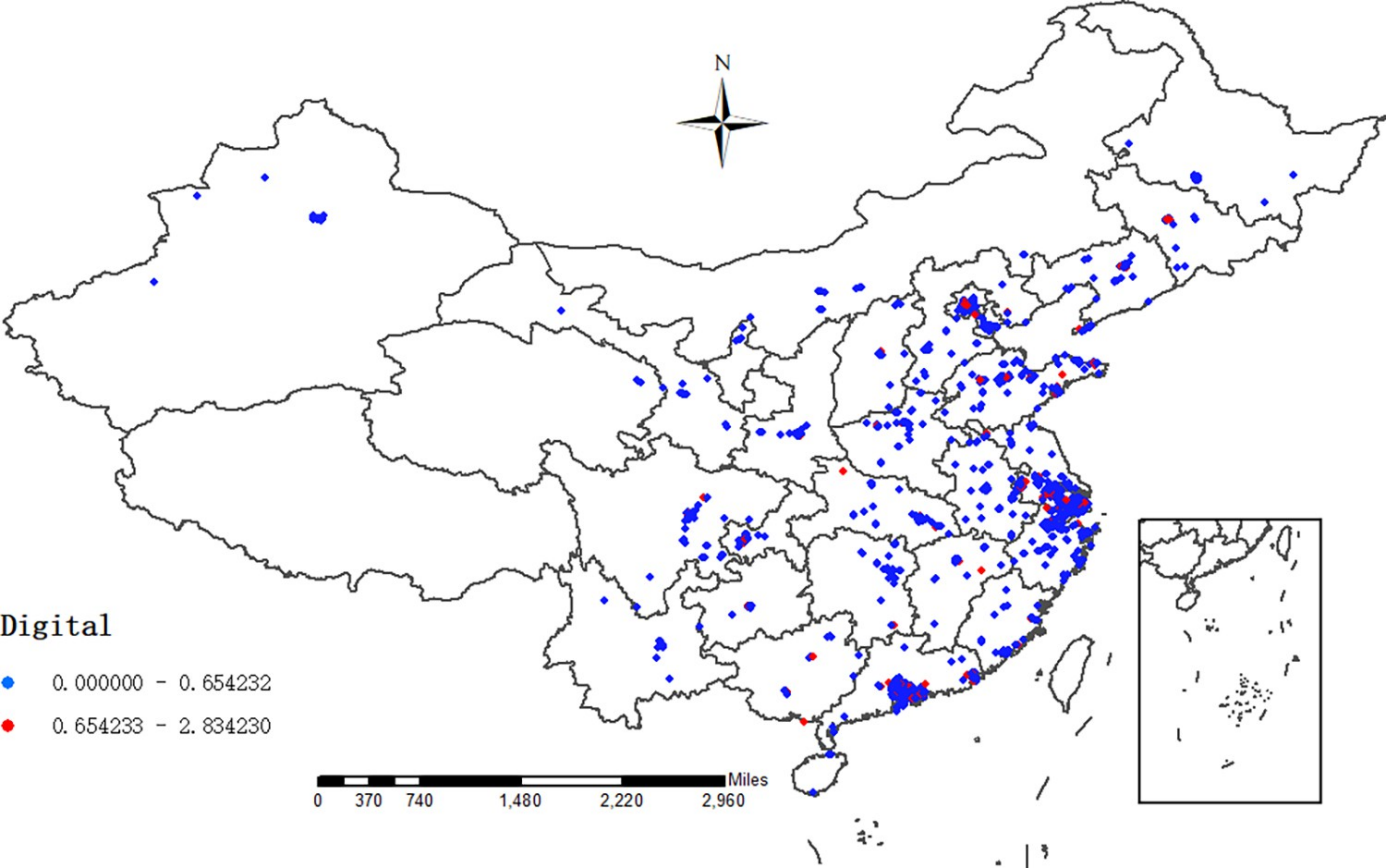
Distribution of digital transformation of listed companies (2018). Note: when the digital transformation of an enterprise is higher than the median value, it is an enterprise with high digital degree, which is represented by red dots in the map and others by blue dots.

Enterprise digital transformation (‘digital’) is the core explanatory variable of this paper. As for the measurement of digital transformation, the current literature mainly focuses on the macro level, such as regional digital transformation, industrial digital transformation and national digital transformation, and it rarely addresses the micro enterprise level. In addition, for the measurement of micro enterprise digital transformation, the current study mostly uses the proportion of intangible assets related to digital transformation [59]. The proportion of enterprise information technology personnel [30] and the application of ERP system [56] are then measured. Most of these indicators have different degrees of defects and deficiencies, thus it is difficult to completely and accurately measure the enterprise digital transformation. To overcome this burden, we use the text analysis method based on machine learning initiated by Yuan et al. [48] for reference to construct the index. The specific steps are as follows: The first step is to construct the enterprise digital term dictionary. Then, 28 important national level digital economy documents from 2010 to 2018 are screened, and multiple rounds of segmentation and deduplication are carried out through python. After expert judgment and discussion, 300 words related to enterprise digital transformation are determined. Next, the frequency of 300 words related to enterprise digital transformation in 28 documents is counted again, and the words with a retention frequency of more than or equal to 5 times are retained. Finally, 180 words related to digital transformation are obtained, which constitute the digital term dictionary of this paper. The second step is the text analysis of the relevant paragraphs of the annual report. The annual reports of A-share listed companies from 2010 to 2018 are downloaded from cninfo.com, the management discussion and analysis (MD & A) part is extracted, and the total number of words in this part and the frequency of 180 digital words are counted. The third step is to build enterprise digital transformation indicators. The sum of the frequency of digital words in each company’s annual report is divided by the total length of MD & A paragraphs. For the ease of expression, this index is expanded 100 times to obtain the enterprise digital transformation index. The higher the index, the higher the degree of enterprise digital transformation. The figure below (Fig 1) depicts the regional distribution of digital transformation of Listed Companies for 2018. It is clearly seen that enterprises with high digital degree (red dot) are mainly scattered in the eastern coastal area, while those with low digital degree (blue dot) are concentrated in the eastern and central areas.

#### Control variables

Referring to the practices of Qi et al, He and Liu, Guo, Ye and Xu, and Wang [17, 55, 60–62], other variables are added to control, such as enterprise size (‘Size’), enterprise age (‘Age’), enterprise value (‘Tobin-Q’), asset liability ratio (‘Debt’), degree of marketization (‘Market’), nature of property right (‘Soe’) and environmental responsibility (‘Duty’). On the one hand, the aim is to enhance the explanatory power of the model. On the other hand, we intend to reduce the endogenous problems caused by missing variables as much as possible. The main variables involved in this paper are summarized in Table 1.

**Table 1:** Main variables.

Variable name	Variable meaning	Calculation method
Green	green innovation	number of green authorized patents / total number of patent applications
Digital	degree of digital transformation	text analysis
Size	enterprise size	log (total assets +1)
Age	enterprise age	log (time of establishment +1)
Tobin-Q	enterprise value	market value / capital replacement cost
Debt	asset liability ratio	total liabilities / total assets
Market	degree of marketization	excerpt from China’s provincial marketization index report
Gdp	degree of economic development	log (province actual GDP+1)
Soe	nature of property right	1 for state-owned and 0 for others
Duty	environmental responsibility	excerpt from social responsibility report of Hexun listed company

#### Descriptive statistics

The descriptive statistics of the main variables are summarized in Table 2. It is not difficult to see that the average share of granted green patents of enterprises is 0.066, which is higher than the average of 0.015 calculated by Qi et al. [55]. The possible reason is that the data sampling interval is different. The data sampling period of Qi et al. [55] is 1990–2010, and the data sampling period of this paper is 2010–2018. It is well known that, in recent years, the state has paid increasing attention to technological innovation and ecological environmental protection, resulting in a rising proportion of granted green patents by enterprises. The average value of digital transformation degree (Digital) is 0.276, which is very close to the calculation result of Yuan et al. [48] at 0.226, indicating that the calculation result of the present paper is credible.

**Table 2:** Descriptive statistics.

Variable	Obs	Mean	Std. Dev.	Min	Max
Green	7625	0.066	0.148	0	0.947
Digital	7625	0.276	0.340	0	2.834
Size	7625	22.020	1.301	19.863	26.186
Age	7625	1.766	0.895	0	3.219
Tobin-Q	7625	2.097	1.193	0.893	8.044
Lev	7625	0.389	0.201	0.049	0.870
Market	7625	10.803	2.368	4.409	14.149
Gdp	7625	11.300	0.491	9.948	12.153
Soe	7625	0.326	0.469	0	1
Duty	7625	0.436	1.010	0	3.178

Note: The author calculated it manually.

#### Empirical equation

In order to investigate the impact of enterprise digital transformation on green innovation in a general sense, the following two-way fixed effect model is constructed with reference to the practices of Aghion et al., Zhang and Liu [63, 64]:

$${\mathrm{Green}}_{\mathrm{it}}=\beta_{0}+\beta_{1}{\mathrm{Digital}}_{\mathrm{it}}+\beta_{2}{Z1}_{\mathrm{it}}+\beta_{3}{Z2}_{\mathrm{pt}}+u_{i}+\lambda_{t}+\varepsilon_{\mathrm{it}}$$ (1)

Among the variables, *Green*_*it*_ represents the enterprise green innovation, which is measured by the proportion of enterprise green granted patents; subscript *t* represents the year, *i* represents the enterprise and *p* represents the province; *Digital*_*it*_ represents the digital transformation of enterprises, which is constructed by the text analysis method (see the above for details); *Z1*_*it*_ represents the enterprise level control variable; *Z2*_*pt*_ represents the provincial level control variable; *u*_*i*_ represents the fixed effect of enterprises to solve the problem of missing variables that do not change over time but vary from individual to individual; *λ*_*t*_ represents a time fixed effect to solve the problem of missing variables that do not change with individuals but vary with time; *ε*_*ipt*_ is an error term, which uniformly adopts the robust standard error clustered to provinces; *β*_*1*_ denotes the key coefficient of this paper, which is expected to be significantly positive, that is, the digital transformation of enterprises promotes green innovation, so as to effectively crack the Solow productivity paradox.

## Empirical results

### Basic regression

Using the method of stepwise regression, the impact of enterprise digital transformation on green innovation is observed, and the specific results are shown in Table 3. The basic regression results are as follows: Column (1) lists the regression results without any control. Enterprise digital transformation significantly promotes green innovation, but the problem of missing variables may occur; Column (2) is the regression result after the introduction of control variables. The digital transformation of enterprises has a positive impact on green innovation, but the result is no longer significant, and the individual heterogeneity of enterprises may be omitted; Column (3)—when further controlling the regression results of enterprise fixed effect, enterprise digital transformation has a significant positive role in promoting green innovation, indicating that the previous enterprise individual heterogeneity seriously interferes with the marginal impact of enterprise digital transformation; Column (4)—the further control of regression results of time fixed effect leads to enterprise digital transformation still having a significant positive promoting effect on green innovation, and the coefficient decreases slightly, indicating that the effect of time heterogeneity is small; Column (5)—further using the robust standard error clustered to the provincial level for regression to eliminate the correlation of error terms among provinces, the enterprise digital transformation still has a significant positive role in promoting green innovation, and the change of standard error is very small. To sum up, the empirical analysis results fully show that the digital transformation of enterprises has significantly promoted the level of green innovation, which provides direct evidence at the micro enterprise level for cracking the Solow productivity paradox.

**Table 3:** Basic regression.

	(1)	(2)	(3)	(4)	(5)
VARIABLES	Green	Green	Green	Green	Green
Digital	0.0107**	0.00832	0.0521***	0.0470***	0.0470***
	(0.00500)	(0.00525)	(0.0115)	(0.0116)	(0.0115)
Controls	N	Y	Y	Y	Y
Firm effect	N	N	Y	Y	Y
Year effect	N	N	N	Y	Y
Cluster region	N	N	N	N	Y
Observations	7,625	7,625	7,625	7,625	7,625
R-squared	0.001	0.022	0.016	0.023	0.023

Note

*, * *, * * * represent 10%, 5% and 1% respectively. The robust standard error of clustering to provinces is used in parentheses. Controls represents control variables. See the above for details. Firm effect represents enterprise fixed effect, year effect represents year fixed effect, and cluster region represents clustering standard error.

### Robustness check

In order to ensure the robustness of the basic regression results mentioned above, a series of robustness analysis are carried out.

#### Proportion of green patent applications

Wang et al., Li and Zheng [62, 65] considered that grants of green patent technology require detection and the payment of an annual fee, and it is vulnerable to political intervention, making it unstable and uncertain. Moreover, green patents have been implemented in the process of application, which has an impact on enterprise performance, so as to more reliably and timely reflect the real green technology innovation ability of enterprises. Therefore, this paper uses the proportion of enterprise green patent applications to replace the original core explanatory variable. The regression results are shown in column 4 (1) below. It is obvious that enterprise digital transformation has a significant positive role in promoting green innovation, the coefficient is slightly reduced, and the result is stable.

#### Number of granted green patents

The reason why this paper uses the proportion of green granted patents as the core explanatory variable is to eliminate the impact of enterprise scale and other unobservable factors on enterprise green innovation. However, some scholars [61, 66] still used the absolute number of green granted patents (logarithm) to measure green innovation. To ensure the robustness of results, this paper uses the latter to regress. The results are shown in column 4 (2) of the table below. It is clearly seen that the digital transformation of enterprises has a significant positive role in promoting green innovation. The results are stable, but the coefficient increases greatly, mainly due to the change of dimension.

#### Digital transformation standardization

Considering that there may be great differences between different dimensions of digital transformation-related vocabulary (big data, cloud computing, artificial intelligence, Internet of things, etc.), which will affect the capture of enterprise digital transformation ability, with reference to the practices of Yuan et al. [48], we standardize the digital transformation sub indicators of each level, eliminate the dimensions, and then sum up to form new digital transformation indicators for regression, The results are shown in column 4 (3) of the table below. Clearly, the digital transformation of enterprises has a significant positive role in promoting green innovation, and the results are stable.

#### Principal component method

Considering that the direct digital aggregation of different dimensions mentioned above may be too rough, the principal component analysis method is used to retain the factors with eigenvalues higher than 1, form a new enterprise digital transformation index, and carry out regression again. The results are shown in column 4 of Table 4 below; the enterprise digital transformation has a significant positive role in promoting green innovation, and the results are stable.

**Table 4:** Robustness check.

	(1)	(2)	(3)	(4)	(5)	(6)
VARIABLES	Green2	Patent	Green	Green	Green	Green
Digital	0.0373*	0.175***			0.0470***	0.0516***
	(0.0200)	(0.0464)			(0.0108)	(0.0107)
						(0.0114)
Digital_std			0.0342**			
			(0.0138)			
Digital_pca				0.0119*		
				(0.00650)		
Controls	Y	Y	Y	Y	Y	Y
Firm effect	Y	Y	Y	Y	Y	Y
Year effect	Y	Y	Y	Y	Y	Y
Cluster region	Y	Y	Y	Y	Y	Y
Observations	7,301	7,625	7,625	7,625	7,625	7,625
R-squared	0.036	0.066	0.021	0.021	0.0229	0.033

Note

*, * *, * * * represent 10%, 5% and 1% respectively. The robust standard error of clustering to provinces is used in parentheses. Controls represents control variables. See the above for details. Firm effect represents enterprise fixed effect, year effect represents year fixed effect, and cluster region represents clustering standard error.

#### D-K standard error

Taking into account that the errors in the econometric model may have three problems, namely, heteroscedasticity, autocorrelation and cross-section correlation, this paper re-regresses to obtain the Driscoll-Kraay standard error, that is, the robustness standard error of heteroscedasticity sequence correlation cross-section correlation. Compared with the clustering robust standard error, the former can better control the influence of cross-section correlation. The results are shown in column 4 (5) of the following table. It is obvious the digital transformation of enterprises has a significant positive role in promoting green innovation, and the results are stable.

#### Time trend for provinces and industries

Considering that there may be time change trends in different provinces and industries, if these are related to the digital transformation of enterprises, it may cause serious endogenous problems. For example, some provinces and industries will accelerate the digital transformation of internal enterprises due to the conducive digital policy environment, resulting in errors in the estimation results. Therefore, the provincial time trend and industry time trend are introduced into the control variables and re-regressed. The results are shown in column 4 (6) of the table below. Obviously, the enterprise digital transformation has a significant positive role in promoting green innovation, and the results are stable.

### Enterprise strategic behavior

We must acknowledge that there may be some strategic behaviors that enterprises perform to interfere with regression results in order to gain more policy benefits from digital transformation. The construction of enterprise digital transformation indicators is mainly based on the information disclosed in the enterprise annual report, which may have problems such as strategic speculation or exaggerated suspicion [67]. These result in a large deviation in the degree of digital transformation of some enterprises, which may affect the reliability of regression results. To eliminate the potential impact of enterprise strategic behavior, we mainly apply the practices of Yuan et al. [48] and remove some enterprise samples for reinspection. The specific results are shown in Table 5 below. Column (1)—considering that most companies listed on the China Growth Enterprise Market (CGEM) belong to high-tech enterprises, and there are many connections with the Internet and Internet business model that are prone to extreme impact, it is straightforward that the digital transformation of enterprises plays a significant positive role in promoting green innovation by removing them and returning them again. Column (2)—considering that some enterprises may underestimate the impact, for example, the digital transformation of some enterprise samples is 0, which may be caused by the failure of enterprises to disclose information in time, it easy to find that the digital transformation of enterprises has a significant positive role in promoting green innovation. Column (3)—considering that enterprises may be suspected of exaggerating the disclosure times of digital words in exchange for supporting national policies, it is clear that the digital transformation of enterprises plays a significant positive role in promoting green innovation by referring to the practice of Zhao et al. [67] and eliminating the residual values of 80th percentile and above. Column (4)—considering that the companies that manipulate the vocabulary related to enterprise digital transformation are most likely those that have been punished by China Securities Regulatory Commission or Stock Exchange due to information disclosure requirements, it is easy to establish that enterprise digital transformation has a significant positive role in promoting green innovation by removing the sample and returning it again; Column (5)—considering that simply eliminating the enterprises punished for information disclosure may not be completely straightforward, it is obvious that the digital transformation of enterprises plays a significant positive role in promoting green innovation when only retaining the samples with good or excellent evaluation results of information disclosure on stock exchanges.

**Table 5:** Enterprise strategic behavior check.

	(1)	(2)	(3)	(4)	(5)
VARIABLES	Green	Green	Green	Green	Green
Digital	0.0685***	0.0459***	0.00972*	0.0589***	0.0365***
	(0.0155)	(0.0114)	(0.00516)	(0.0146)	(0.0121)
Controls	Y	Y	Y	Y	Y
Firm effect	Y	Y	Y	Y	Y
Year effect	Y	Y	Y	Y	Y
Cluster region	Y	Y	Y	Y	Y
Observations	5,694	7,350	6,100	5,628	4,701
R-squared	0.032	0.023	0.020	0.026	0.019

Note

*, * *, * * * represent 10%, 5% and 1% respectively. The robust standard error of clustering to provinces is used in parentheses. Controls represents control variables. See the above for details. Firm effect represents enterprise fixed effect, year effect represents year fixed effect, and cluster region represents clustering standard error.

### Semiparametric regression

**Fig 2:**
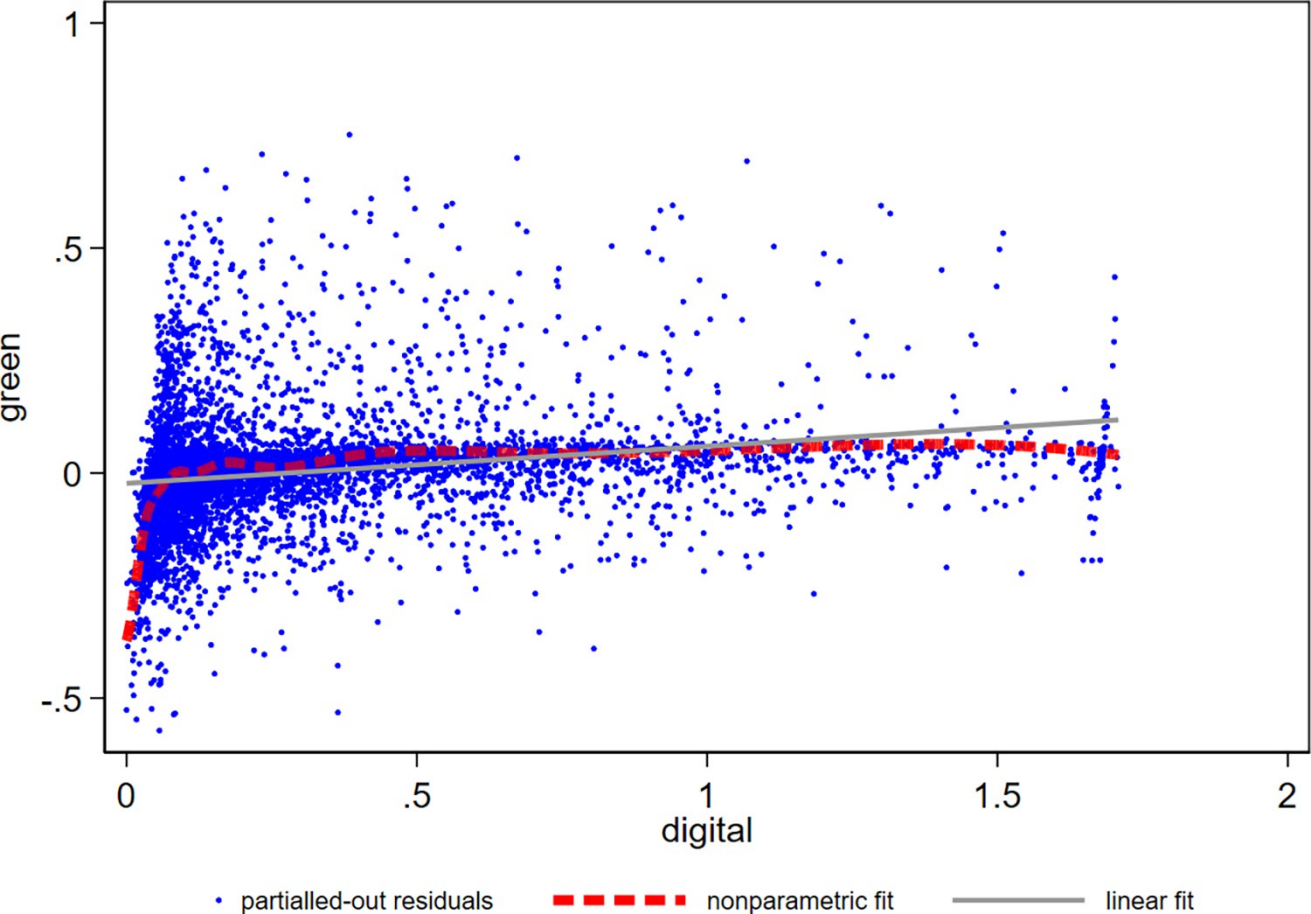
Nonparametric matching of the impact of enterprise digital transformation.

Some studies have pointed out that the impact of enterprise digital transformation on innovation performance may be nonlinear, such as an inverted U-shaped or uncertain relationship [29, 68, 69]. The existing researches on the impact of digital nonlinearity basically involve parametric regression, that is, the form of regression equation has been assumed in advance, which may lead to potential model setting errors. If nonparametric regression is used, although the error of model setting can be avoided, it is easy to cause dimensional disaster. Therefore, based on comprehensive consideration, this paper decides to use semiparametric regression, that is, it does not assume the specific function expression of the core explanatory variable digital, therefore, it can more truly reflect the impact of enterprise digital transformation on green innovation from a data-driven perspective, as follows:

$${\mathrm{Green}}_{\mathrm{it}}=\beta_{0}+\beta_{1}{F(\mathrm{Digital}}_{\mathrm{it}})+\beta_{2}{Z1}_{\mathrm{it}}+\beta_{3}{Z2}_{\mathrm{pt}}+u_{i}+\lambda_{t}+\varepsilon_{\mathrm{it}}$$ (2)

where *F*(*Digital*_*it*_) represents the function of enterprise digital transformation. The specific form is uncertain. The meanings of other variables are the same as above and will not be repeated here. B-splines is used for nonparametric matching. The specific results are shown in Fig 2. From the nonparametric matching curve (dashed dash line), it is easily seen that, when the degree of enterprise digital transformation is low, the marginal impact on green innovation is considerable. With the increase of the digital transformation degree, the marginal impact decreases rapidly until it reaches near 0, showing typical nonlinear characteristics. In comparison, the linear matching curve (solid line) indicates that the average marginal impact of enterprise digital transformation on green innovation is positive, and the conclusion is consistent with the basic regression result.

## Endogeneity

In order to ensure the stability of the basic regression results, although a series of robustness tests have been carried out, the enterprise digital transformation and green innovation are still subject to obvious endogenous challenges. Firstly, the improvement of enterprise digital transformation will promote green innovation. Secondly, after the enterprise’s green innovation level is improved, obtaining excess profits will further encourage enterprises to increase investment in digital technology innovation, promote the deep integration of digital technology and production and operation processes, and grab higher digital dividends. To deal with these potential endogenous problems, this paper uses the instrumental variable method and the difference in difference method for effective mitigation.

### Instrumental variable method

Referring to the practices of Zhao et al. [67] and Zhou et al. [18], the intersection of the number of Internet users in China lagging behind the first period and the number of fixed phones per 10,000 people in various cities in 1984 is used as the instrumental variable of enterprise digital transformation in the current period. On the one hand, with the continuous development of traditional communication technology, the telecommunications infrastructure in local history will affect the application of Internet technology in the subsequent stage at the technical level and in terms of usage habits, so as to meet the relevance. On the other hand, the impact of traditional telecommunications tools such as fixed telephone on economic development gradually decreases with the decline of use frequency, which meets the exclusivity. Consequently, the two-stage least square method is used for regression, and the specific results are shown in Table 6, column (1) below. It is established that the enterprise digital transformation has a significant role in promoting green innovation, and it has passed the unidentifiable and weak identification tests. Column (2) comprises the result of using the proportion of enterprise green patent applications as the explanatory variable regression. Enterprise digital transformation still has a significant role in promoting green innovation, and it passes the unidentifiable and weak identification tests.

**Table 6:** Instrumental variable method.

	(1)	(2)	(3)	(4)
VARIABLES	Green	Green2	Green	Green2
Digital	0.0328**	0.0188***	0.0197*	0.0195***
	(0.0141)	(0.00628)	(0.0103)	(0.00702)
Controls	Y	Y	Y	Y
Firm effect	Y	Y	Y	Y
Year effect	Y	Y	Y	Y
Cluster region	Y	Y	Y	Y
Unidentifiable test	27.072	27.072	27.940	27.940
Weak identification test	23.166	23.166	24.507	24.507
Observations	6,372	6,372	7,056	7,056
R-squared	0.026	0.017	0.011	0.015

Note

*, * *, * * * represent 10%, 5% and 1% respectively. The robust standard error of clustering to provinces is used in parentheses. Controls represents control variables. See the above for details. Firm effect represents enterprise fixed effect, year effect represents year fixed effect, and cluster region represents clustering standard error. The unidentifiable test reports Kleibergen-Paap rk LM statistics, and the corresponding p value is 0. Obviously, the unidentifiable original hypothesis is strongly rejected, indicating that there is a correlation between instrumental variables and endogenous variables. Weak identification test reports Kleibergen-Paap rk Wald F statistic, which is much greater than the threshold corresponding to 10% level of Stock-Yogo weak identification test (the thresholds corresponding to 10%, 15% and 20% are 16.38, 8.96 and 6.66 respectively), indicating that instrumental variables have strong correlation with endogenous variables.

Referring to the practice of Guo and Luo [70], the Internet data of the first lag period is used as the instrumental variable, that is, the Internet penetration rate of each province from 2001 to 2009 is employed as the instrumental variable, and then the two-stage least square method is used for regression. The specific results are shown in Column (3) of Table 6 below. Clearly, the digital transformation of enterprises can significantly promote green innovation and pass the unidentifiable and weak identification tests. Column (4) is the result of regression using the proportion of enterprise green patent applications as the explanatory variable. Enterprise digital transformation still plays a significant role in promoting green innovation and passes the unidentifiable and weak identification tests.

### Policy pilot of industrialization and informatization zones

#### Policy background and basic regression

In 2013, Zhejiang Province initiated the construction of informatization and industrialization integration demonstration zone (hereinafter referred to as "two zones") in accordance with the national deployment. It has since carried out dynamic tracking, questionnaire surveys and performance evaluation for enterprises participating in digital transformation each year. In 2014, it issued the implementation opinions on building a national demonstration zone with the deep integration of informatization and industrialization, which is leading, pioneering and exemplary and can provide empirical evidence and policy enlightenment for other provinces in China [5]. After the implementation of the policies of the two zones in Zhejiang Province, enterprises in the province will have a suitable digital business environment and benefit from digital related supporting facilities and incentive policies, which is bound to accelerate the digital transformation of enterprises. Therefore, the policies of the two chemical zones are closely related to the digital transformation of enterprises and are not adversely affected by the green innovation of enterprises. This provides a convenient quasi-natural experiment for this paper to effectively identify the causal relationship between enterprise digital transformation and green innovation. In order to test the impact of policy shocks in the two regions, the following measurement models are set:

$${\mathrm{Green}}_{\mathrm{it}}=\beta_{0}+\beta_{1}{\mathrm{Post}}_{t}\#{\mathrm{Treat}}_{i}+\beta_{2}{Z1}_{\mathrm{it}}+\beta_{3}{Z2}_{\mathrm{pt}}+u_{i}+\lambda_{t}+\varepsilon_{\mathrm{it}}$$ (3)

Among the variables, *Post*_*t*_ represents the dummy variable for the experimental period; when the year is 2014 or later, the value is 1, otherwise it is 0; *Treat*_*i*_ represents the dummy variable for the experimental group; when the enterprise belongs to Zhejiang Province, its value is 1, otherwise it is 0; the explanation of other variables is the same as above. We pay attention to the coefficient *β*_*1*_ and expect it to be significantly positive, indicating that the policy impact of the two areas in Zhejiang Province has a beneficial effect, that is, the digital transformation of enterprises has a significant effect on green innovation. The specific regression results are shown in Table 7 below. The results of column (1) are significantly positive, indicating that the two area policies have significantly improved the level of green innovation of the enterprise; column (2) is the replacement of the experimental group dummy variable with the digital transformation of the enterprise, which is essentially intensity DID that can fully reflect the impact of different companies’ digital transformation levels on green innovation, and the result is still significantly positive; column (3) uses the proportion of green patent applications as the explained variable, and the coefficient is significantly positive; column (4) is the regression of the usage intensity on the proportion of green patent applications, and the coefficient is still significant and positive; column (5) is the regression using PSM-DID, and the purpose is to enhance the matching accuracy between the experimental group and the non-experimental group by eliminating abnormal samples. It is easy to find that the coefficient is still significant. The above results comprehensively show that the digital transformation of enterprises has significantly promoted green innovation.

**Table 7:** Difference in difference results.

	(1)	(2)	(3)	(4)	(5)
VARIABLES	Green	Green	Green2	Green2	Green
c.treat#c.post	0.0296***		0.0592**		0.05**
	(0.00869)		(0.0234)		(0.025)
c.digital#c.post		0.0611**		0.138*	
		(0.0272)		(0.0705)	
Controls	Y	Y	Y	Y	Y
Firm effect	Y	Y	Y	Y	Y
Year effect	Y	Y	Y	Y	Y
Cluster region	Y	Y	Y	Y	N
Observations	7,668	7,668	7,668	7,668	7668
R-squared	0.044	0.046	0.080	0.082	0.030

Note

*, * *, * * * represent 10%, 5% and 1% respectively. The robust standard error of clustering to provinces is used in parentheses. Controls represents control variables. See the above for details. Firm effect represents enterprise fixed effect, year effect represents year fixed effect, and cluster region represents clustering standard error.

#### Parallel trend test

As widely known, Zhejiang Province, as the vanguard of China’s digital economic development, has been at the forefront of digital development. Therefore, the central government’s selection of this province as the pilot of the two industrialization zones may violate the principle of randomness, resulting in differences between the experimental group and the non-experimental group before the implementation of policies, which violates the parallel trend hypothesis. Although the PSM method is used for the above mitigation, in order to ensure the robustness of the results, a dynamic test is conducted below to ensure that the model meets the premise assumption of parallel trend. The measurement model is set as follows:

$${\mathrm{Green}}_{\mathrm{it}}=\beta_{0}+\beta_{i}\sum_{i=2010}^{2018}{\mathrm{Treat}}_{i}\#{\mathrm{Post}}_{t}+\beta_{2}{Z1}_{\mathrm{it}}+\beta_{3}{Z2}_{\mathrm{pt}}+u_{i}+\lambda_{t}+\varepsilon_{\mathrm{it}}$$ (4)

**Fig 3:**
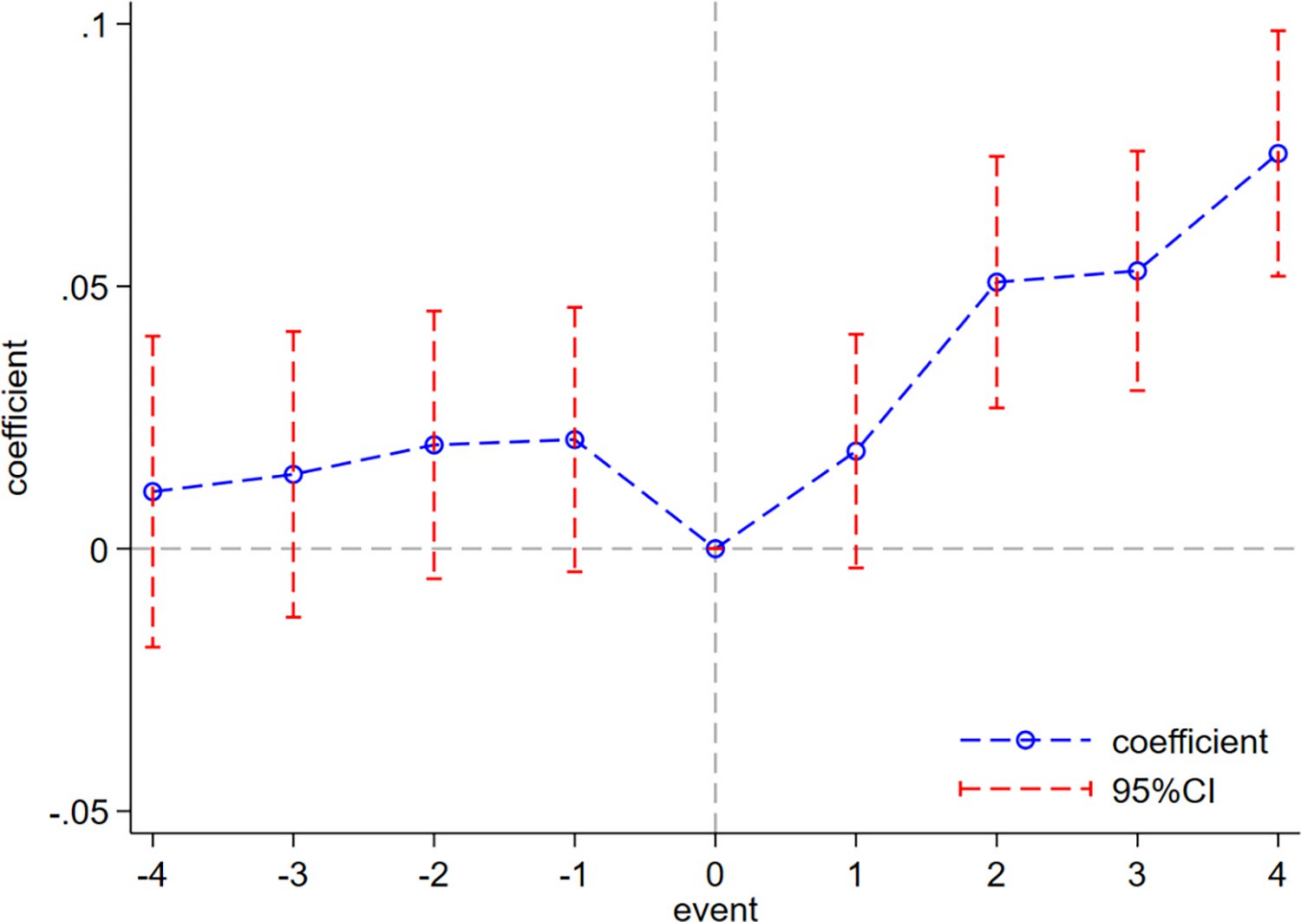
Dynamic test.

Among the variables, $\sum_{i=2010}^{2018}{\mathrm{Post}}_{t}$ represents the dummy variable for each year, with 2014 set as the base period, and the other variables are explained as above. The specific regression results are shown in Fig 3, which reveal that, before the policy implementation, the difference between the experimental group and the control group gradually increased but was not significantly different from 0 within the 95% confidence interval, indicating that the experimental group before the implementation of the policy meets the assumption of parallel trend with the control group. In the first year after the policy implementation, the effect of the policy was not obvious, and then it showed a gradual upward trend, indicating that the impact of the two industrialization zone policies on the green innovation of enterprises was gradually increasing.

#### Placebo test

**Fig 4:**
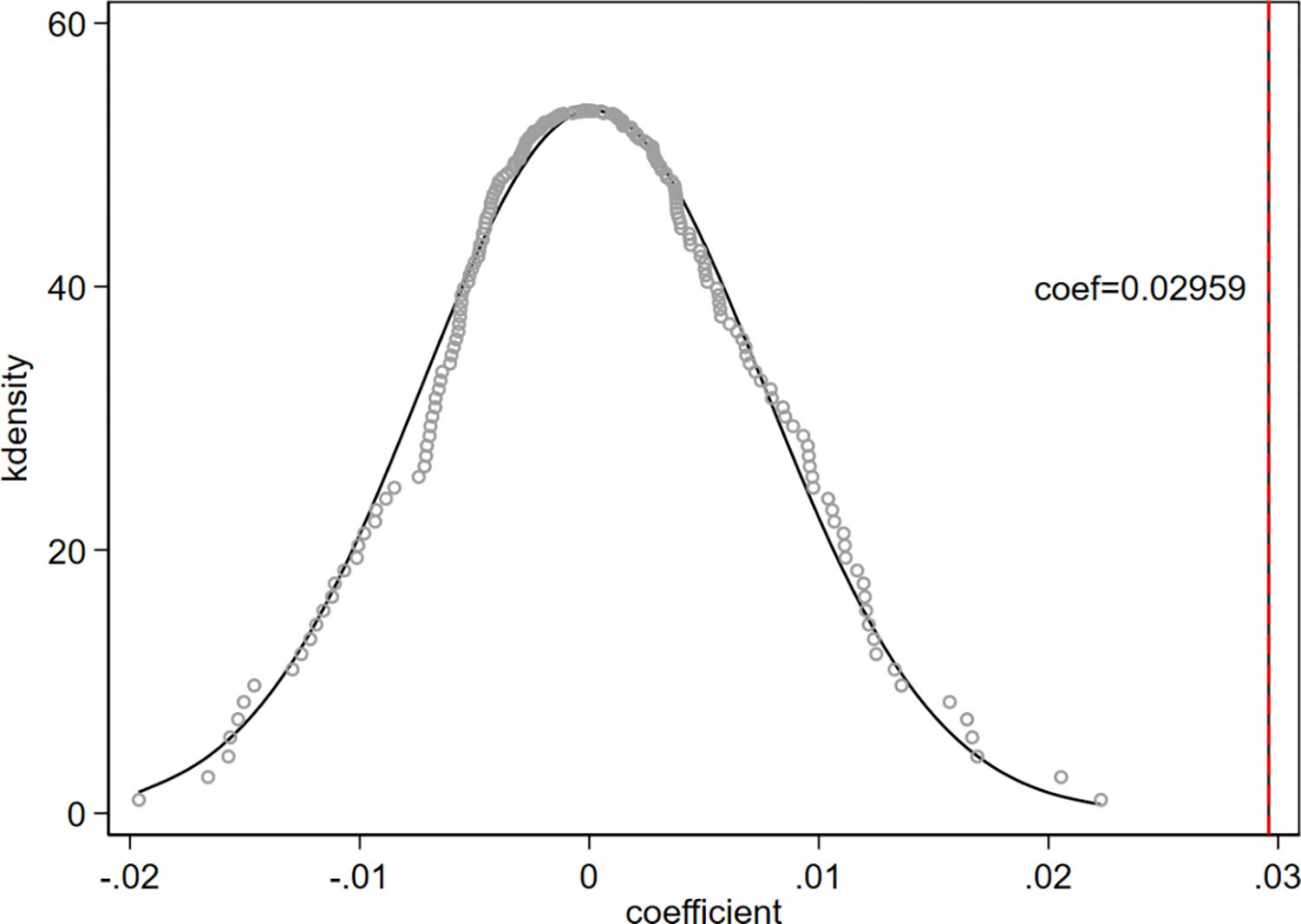
Placebo test.

In order to further ensure the robustness of the regression results, a placebo test is also implemented, that is, assume that the policy occurs in any province, and then construct a false experimental group for regression. After 200 regressions by random sampling, the density distribution of the coefficients is shown in Fig 4 below. As can be seen from the figure, the coefficients of 200 false experiments show a normal distribution and are concentrated near the value of 0, which is significantly different from the coefficients of the basic regression results. This fully reveals that the above regression results are indeed caused by the real urbanization policy in Zhejiang Province rather than other unobservable factors.

## Mechanism test

### Improvement of information processing capability

Previous research has demonstrated that enterprise digital transformation can promote green innovation by improving the enterprise information processing capacity. There are two main methods in the current literature to measure the enterprise information processing ability: the first one is to conduct field research on enterprises and then obtain first-hand data, which is not suitable for this paper; the second one includes judging whether the enterprise uses the integrated management system (OA, ERP, HRM, and other systems). However, because the integrated management system itself embodies a digital technology application and has been included in the enterprise digital transformation calculation process, there is a pseudo regression problem of self-interpretation. This paper uses three indicators to indirectly represent the enterprise’s information processing capacity; the first one is knowledge (‘Knowledge’). Because the information processing capacity is enhanced, the enterprise will obtain more accurate and valuable information knowledge, which means that the enterprise’s knowledge capital stock will be greatly increased. Here, we refer to the practices of Cheng and Lu [71] and use the proportion of R&D personnel to measure the knowledge capital. The reason is that the creation, absorbtion and application of enterprise knowledge capital will eventually fall on people, so the improvement of knowledge capital is directly reflected in the improvement of human capital. The second indicator is the quality management system certificate score (‘Quality’), because the enterprise digital transformation promotes the enterprise to realize the information monitoring and big data processing of the whole production process, which will improve the quality management level of the entire process, and then elevate the quality management system certificate score. The third indicator is the environmental management system certification score (‘Greenman’), as the digital transformation of enterprises can also realize the information monitoring and data analysis of energy consumption and pollution emission in the production process of enterprises. Under the external pressure of environmental regulation and the environmental awareness of green production, enterprises can reduce their energy consumption by optimizing business processes, enhancing production modes, reducing pollution and improving the certification score of the environmental management system. The data of quality management system certificate score and environmental management system certification score are derived from the social responsibility report of Hexun-listed companies for logarithmic processing. The specific regression results using the three indicators are shown in columns (1)—(3) of Table 8 below. Clearly, the enterprise digital transformation can significantly promote the enterprise knowledge capital, the score of quality management system certificate, and the score of environmental management system certification, indicating that the enterprise digital transformation improves the information processing ability. Therefore, Hypothesis 1 is proved.

**Table 8:** Mechanism test.

	(1)	(2)	(3)	(4)	(5)	(6)
VARIABLES	Knowledge	Quality	Greenman	MC	Posi	Sep
Digital	1.249**	0.0635***	0.105***	-0.0701***	-0.00124***	0.0167**
	(0.569)	(0.0120)	(0.00461)	(0.00467)	(0.000251)	(0.00694)
Controls	Y	Y	Y	Y	Y	Y
Firm effect	Y	Y	Y	Y	Y	Y
Year effect	Y	Y	Y	Y	Y	Y
Cluster region	Y	Y	Y	Y	Y	N
Observations	2,137	15,227	15,227	15,227	15,227	15,227
R-squared	0.105	0.947	0.905	0.0175	0.0184	0.0634

Note

*, * *, * * * represent 10%, 5% and 1% respectively. The robust standard error of clustering to provinces is used in parentheses. Controls represents control variables. See the above for details. Firm effect represents enterprise fixed effect, year effect represents year fixed effect, and cluster region represents clustering standard error.

### Reduction of enterprise costs

According to previous analyses, the digital transformation of enterprises mainly reduces the internal control cost and the external control cost, so as to have excess funds to increase the investment in R&D and innovation, and pollution control equipment, so as to improve the level of green innovation. With regards to the measurement of internal and external costs of enterprises, Li et al. [72] considered that the proportion of enterprise management expenses can directly reflect the internal control costs of enterprises. Zhao et al. [73] indicated that the expenses in enterprise rent-seeking activities are "often included in the secondary business entertainment account in management expenses", which shows that the enterprise management expenses also reflect the external control cost of the enterprise to a certain extent. At the same time, Qin et al. [74] reported that the proportion of sales expenses can measure the external cost of the enterprise. On the whole, this paper decides to use "management expense/operating revenue" (‘MC’) and "sales expense/operating revenue" (‘Posi’) to represent the internal control cost and external control cost of the enterprise, respectively. The specific regression results are shown in columns (4)-(5) of Table 8 below. It can be inferred that the digital transformation of the enterprise has significantly reduced the proportion of management expense and sales expense, which means the reduction of internal and external control costs of the enterprise, thereby hypothesis 2 is considered as proved.

### Improvement of professional division of labor

It has been established that enterprise digital transformation can promote green innovation by improving the professional division of labor. For the measurement of enterprise specialization, this paper refers to Adelman, Buzzell and Yuan et al. [48, 75, 76], and uses the value added to sales method. The basic idea is that the higher the proportion of enterprise added value in sales revenue, the higher the level of enterprise vertical integration, that is, the lower the degree of specialization. The specific calculation is as follows: Sep = (added value—tax net profit + net assets * average return on net assets) / (main business income—after tax net profit + net assets * average return on net assets), in which the added value is expressed by the difference between the enterprise’s sales and the purchase amount, the average return on net assets is measured by the average of the enterprise’s industry in the recent three years, and the purchase amount is calculated by the equation of “(cash paid for purchasing goods and receiving labor services + opening prepayment—closing prepayment + closing accounts payable—opening accounts payable + closing notes payable—opening notes payable) / (1 + VAT rate of purchased goods) + opening inventory—closing inventory”. To facilitate the interpretation of the results, the enterprise’s professional division of labor is treated as “1-Sep”. The specific regression results are shown in column (6) of Table 8 below, which clearly indicate that the digital transformation of enterprises has significantly improved the degree of specialization of enterprises. Consequently, Hypothesis 3 is proved.

## Heterogeneity analysis

Considering the fact that the impact of enterprise digital transformation on green innovation may have differential performance in separate dimensions, this part will conduct a heterogeneity test, and the specific measurement model is set as follows:

$${\mathrm{Green}}_{\mathrm{it}}=\beta_{0}+\beta_{1}{\mathrm{Digital}}_{\mathrm{it}}\#M+\beta_{2}{Z1}_{\mathrm{it}}+\beta_{3}{Z2}_{\mathrm{pt}}+u_{i}+\lambda_{t}+\varepsilon_{\mathrm{it}}$$ (5)

where M represents the dummy variable of enterprise ownership type, the dummy variable of enterprise political connection, the intensity of provincial environmental regulation, enterprise life cycle, regional dummy variable and enterprise social responsibility score, respectively. The specific meaning of the variable is shown below.

### Ownership type

Zhong and Yang [57] considered that, compared with general technological innovation, green technological innovation has higher public value attribute, and its private rate of return is significantly lower than its social rate of return. State-owned enterprises are an important tool for the government to make up for market failure and solve the problem that the private rate of return is lower than the social rate of return [77]. At the same time, state-owned enterprises undertake more tasks of energy conservation and emission reduction and thus face greater pressure on these tasks. Therefore, compared with private enterprises, state-owned enterprises are likely to engage in less profitable green technology innovation. Thus, in the sample of state-owned enterprises, the promotion effect of enterprise digital transformation on green innovation is expected to be greater. Here, the enterprise registration type is used for judgment. If it is a state-owned enterprise, its value is 1, otherwise it is 0. The specific regression results are shown in column (1) of Table 10 below. it is clearly seen that the coefficient of interactive term (‘c.Digital#c.Soe’) is significantly positive, indicating that enterprise digital transformation is more conducive to promoting green innovation in the sample of state-owned enterprises, which is in line with the expectations.

### Political connection

If an enterprise can establish close political ties with the government, it will be easier for this enterprise to obtain national legal and policy support and enjoy more preferential treatment in the factor market and product market. This phenomenon in turn will greatly weaken the policy uncertainty and external risks faced in the innovation process [78], At the same time, it also weakens the external competitive pressure brought by the positive spillover effect of innovation. Meanwhile, Yao [79] suggested that political connection makes local governments absent in the supervision and implementation of social responsibility, it inhibits the effectiveness of environmental regulation by the central government, and leads to the low environmental performance of enterprises. Lei et al. [80] also reported that political connection makes the government reduce the punishment on enterprises, resulting in these enterprises having no fear in the process of pollution emission. Thus, political connection is unlikely to have any obvious regulatory effect on the relationship between enterprise digital transformation and green innovation. To determine whether the enterprise has political connection, if the chairman or general manager is a section level cadre, it is assigned 1, for a department level cadre, it is 2, 3, or 4, and others are assigned 0. The specific results are shown in column (2) of Table 10 below. Obviously, the coefficient of interaction term (‘c.Digital#c.Political’) is negative, however, the statistics are not significant, indicating that the impact of enterprise digital transformation on green innovation is not regulated by political relevance, which is in line with the expectations.

### Environmental regulation

The stricter the environmental regulation, the more enterprises are subjected to external political pressure, environmental protection pressure, and social responsibility pressure. According to the Porter hypothesis, this will force enterprises to strengthen R&D innovation and improve productivity to make up for additional environmental costs, so as to realize the double achievement of environmental performance and innovation performance improvement. Therefore, it is expected that the stronger the local environmental regulation, the higher the impact of enterprise digital transformation on green innovation. With regards to measuring the intensity of environmental regulation, the proportion of words related to the environment (including environmental protection, pollution, energy consumption, emission reduction, pollution discharge, ecology, green, low carbon, air, chemical oxygen demand, sulfur dioxide, carbon dioxide, PM10 and PM2.5) in the total word frequency in the government work reports over the years is used to reflect the government’s attention to the environment and the intensity of regulation in those years. The specific results are shown in column (3) of Table 10 below. It is shown that the coefficient of interaction term (c.Digital#c.Regulat) is significantly positive, indicating that where the intensity of environmental regulation is high, the enterprise digital transformation plays a greater role in promoting green innovation, which is in line with the expectation of the Porter hypothesis.

### Enterprise life cycle

According to the enterprise life cycle theory, enterprises in the growth stage usually have a relatively simple organizational structure and low internal control cost. Due to the lack of capital and high innovation risk, however, the enterprise’s green innovation ability is average; with the continuous development and growth of the enterprise, the capital strength is gradually enhanced, the cash flow is abundant, and the enterprise can shoulder high innovation risk and green investment, thereby improving the enterprise’s green innovation ability. As the enterprise enters the end of maturity or recession stage, the organizational structure tends to be bloated and the cost of internal control gradually increases [48]. At this time, the enterprise is more likely in pursuit of stability and risk aversion, which is bound to reduce the investment in green innovation and weaken its level. Therefore, it is expected that with the continuous growth and development of enterprises, the impact of enterprise digital transformation on green innovation will show an inverted U-shaped trend of first rising and then falling, and the average impact will be uncertain. As for the division of enterprise life cycle, the cash flow combination method of Hasan et al. [81] and Dickinson [82] is used for reference, as shown in Table 9 below.

**Table 9:** Division of enterprise life cycle.

Life cycle	Operating cash flow	Investment cash flow	Financing cash flow
Stage1	<0	<0	>0
Stage2	>0	<0	>0
Stage3	>0	<0	<0
Stage4	<0	>0	≤0 or >0
Stage5	Others		

Furthermore, considering that the sample size at different life cycle stages varies greatly, which may bring deviation to the regression results, some life cycles are combined with reference to the practices of Huang et al. [83]. Specifically, enterprises in the introduction period and growth period are unified into enterprises in the growth period, with an attributed value of 1. Enterprises in the recession period and adjustment period are unified into enterprises in the recession period, with a given value of 3, while enterprises in the mature period remain unchanged, having a value of 2. The specific regression results are shown in column (4) of Table 10 below. It can be seen that though the coefficient of interaction term (‘c.Digital#c.Life’) is not significant, but it is negative and largely in line with our expectations.

**Table 10:** Heterogeneity analysis.

	(1)	(2)	(3)	(4)	(5)	(6)
VARIABLES	Green	Green	Green	Green	Green	Green
c.Digital#c.Soe	0.205***					
	(0.0556)					
c.Digital#c.Political		0.0119				
		(0.0116)				
c.Digital#c.Regulat			0.0986***			
			(0.0253)			
c.Digital#c.Life				-0.00175		
				(0.0105)		
c.Digital#c.Area					0.0415***	
					(0.0123)	
c.Digital#c.Respon						0.0204**
						(0.00919)
Controls	Y	Y	Y	Y	Y	Y
Firm effect	Y	Y	Y	Y	Y	Y
Year effect	Y	Y	Y	Y	Y	Y
Cluster region	Y	Y	Y	Y	Y	Y
Observations	7,526	7,526	7,526	7,526	7,526	7,431
R-squared	0.054	0.051	0.053	0.050	0.055	0.052

Note

*, * *, * * * represent 10%, 5% and 1% respectively. The robust standard error of clustering to provinces is used in parentheses. Controls represents control variables. See the above for details. Firm effect represents enterprise fixed effect, year effect represents year fixed effect, and cluster region represents clustering standard error.

### Regional differences

In a general sense, China’s economy is strong in the East and weak in the West, with large regional differences [15, 17]. To encourage the development of the western region, China implemented the western development strategy relatively early, issuing a large number of financial subsidies and preferential policies, and encouraging rapid economic development to narrow the regional gaps. A substantial number of relevant documents have proved that financial subsidies have obvious inhibition effect on innovation and even lead to a significant number of fraudulent cases. At the same time, due to the relatively backward economic development in the western region, the pressure on officials’ GDP assessment and promotion will be higher, such that there is an internal impulse to sacrifice the environment to promote economic development. In addition, the western region is rich in production resources, and the relevant exploitation and development process will inevitably damage the ecological environment. Moreover, there are signs of transferring highly polluting enterprises to the western region from the eastern region, which will increase the pollution in this region. Overall, the level of green innovation in the western region is low. Therefore, this paper expects that, compared with the western region, the promotion effect of enterprise digital transformation on green innovation is stronger in the eastern region. We assigned 1 to the western region, 2 to the central region and 3 to the eastern region. The specific regression results are shown in column (5) of Table 10 below. It is not difficult to see that the coefficient of interactive term (‘c.Digital#c.Area’) is significantly positive, indicating that with the regional promotion from West to East, the promotion effect of enterprise digital transformation on green innovation is enhanced, which is in line with the expectations.

### Social responsibility consciousness

The reason why enterprises pursue innovation is to improve production efficiency and obtain excess profits, which is in line with the essence of capital chasing profits. When enterprises have a strong sense of social responsibility, they will also pay attention to the negative externalities of production during the process of pursuing excess profits, such as the negative impact of production sewage on the ecological environment and the surrounding enterprises and residents. Therefore, they will carry out energy conservation and emission reduction, taking the initiative to shoulder the responsibility of sustainable economic and social development. Accordingly, when the awareness of enterprise social responsibility is high, it is expected that the digital transformation of enterprises will play a greater role in promoting green innovation. The measurement of enterprise social responsibility consciousness is introduced from the professional evaluation system of social responsibility report of Hexun-listed companies. In the process of calculating the weighting of social responsibility score, subdimensions such as product innovation consciousness score and environmental protection consciousness score are considered, thus the enterprise social responsibility consciousness is evaluated comprehensively and scientifically. The specific regression results are shown in column (6) of Table 10 below. Obviously, the coefficient of interactive term (‘c.Digital#c.Respon’) is significantly positive, indicating that with the improvement of enterprise social responsibility awareness, the promotion effect of enterprise digital transformation on green innovation is increasing, which is in line with the expectations.

## Conclusions and policy recommendations

This paper deeply studies the impact of enterprise digital transformation on green innovation, while trying to provide micro-level evidence for solving the SPP. Specifically, the main work is as follows: first, in a theoretical sense, this paper deeply discusses the potential mechanism of the impact of enterprise digital transformation on green innovation. Second, the enterprise digital transformation is described based on text analysis, and then the relationship between digital transformation and green innovation is strictly tested. Third, a series of robustness tests and endogenous discussions are carried out to strictly identify the causal effects, and the potential mechanisms and heterogeneous effects are also tested. Fourth, based on theoretical and empirical analysis, this paper puts forward some policy suggestions to promote enterprise digital transformation and green innovation. The main findings are as follows: (1) Enterprise digital transformation can significantly promote green innovation. With a series of robustness tests, it provides micro-level evidence for effectively cracking the SPP. (2) More rigorous causal effect identification is carried out through the IV method and the DID method, and the basic conclusion remains unchanged. (3) Three potential mechanisms (information processing capacity, internal and external costs, and professional division of labor) are strictly tested to confirm the theoretical hypothesis proposed above. (4) The heterogeneity analysis indicates that the impact of digital transformation on green innovation is greater among the samples of state-owned type, high environmental regulation areas, eastern regions, and high social responsibility awareness.

The theoretical and empirical studies in this paper enrich the existing literature: First, this paper enriches the research related to the impact effects of digital transformation. The current research on the impact effects of digital transformation mainly focuses on the macro level such as countries, provinces, and cities [10, 16], and only one focuses on “green process innovation” at the micro firm level [19], differing from their research perspective, this paper focuses on “green output innovation” (green patents) and uses richer data of listed companies, better digital transformation indicators and more adequate causal identification strategies to greatly enrich the research on the impact effects of digital transformation. Second, this paper enriches the study of the path mechanisms of digital transformation impact. Existing literature considers the potential mechanisms of digital transformation impact mainly transaction costs and resource mismatch [31], organizational empowerment [32], and this paper explores three new potential mechanisms through in-depth theoretical and empirical analysis, which are information processing capability, enterprise costs and professional division of labor. This paper also contributes the study of Wang et al. [53] and Aldieri et al. [84] which regarded the technological diffusion processes as the important channel, and provides microscopic evidence from developing countries. Third, it enriches the research on the influencing factors of green innovation. Existing studies on the influencing factors mainly focus on command-based regulatory policies [34], market-based regulatory policies [35] and firm organizational structures [36, 37]. In the context of digital economy development, this study focuses on digital transformation as an influencing factor and extends the existing studies. Fourth, this paper provides direct evidence and potential pathways from the micro level for effectively deciphering SPP, enriching the current research on the topic of SPP.

Based on the theoretical and empirical analysis results of this paper, the relevant policy suggestions are as follows: First, help enterprises accelerate digital transformation. Improve the market management system and administrative approval process to create a supportive regulatory environment for enterprise digital transformation. The government should timely issue relevant supporting policies to solve various difficulties that enterprises may face in the painful period of digital transformation. Pay attention to the construction and improvement of digital infrastructure, build a digital development platform and widely gather resources from all walks of life. Second, strengthen the green innovation ability of enterprises. The government should constantly improve the associated systems and mechanisms such as green procurement, green product evaluation and certification, the transformation of green technology innovation achievements, prosper the green technology market and increase the financial and tax policy support for green technology innovation, so as to improve the return rate of green technology innovation and stimulate the green technology innovation vitality of enterprises. Third, reduce the internal and external costs of enterprises, promote the professional division of labor in these enterprises, and unblock the role channel between digital transformation and green innovation. The government should pay attention to improving the level of governance, focus on strengthening system reform, and constantly optimize the business and contract environment, with the aim of resolving the contracting and performance risks of enterprises, fundamentally reducing the internal and external costs for enterprises, and fully exploring the potential of market division of labor. Fourth, some pilot implementation of digital transformation policies should be performed. According to the results of heterogeneity analysis, the digital transformation of enterprises has a stronger promotion effect in the provinces with state-owned type enterprises, a high intensity of environmental regulation, eastern provinces, and samples with high awareness of social responsibility. Therefore, the government can try to pilot and promote digital transformation in a local scope, and then gradually expand it to the whole region step-by-step, in order to control various unexpected risks in a timely manner. Fifth, enhance enterprise social responsibility. The government should strengthen the publicity of the concept of green development, so that the concept of environmental protection is deeply rooted in the hearts of people, and enhances the sense of enterprise social responsibility. The government should also strengthen environmental protection supervision and law enforcement, enhance the cost of enterprise pollution emissions, and force enterprises to improve the level of green innovation. At the same time, the government should improve the relevant system and mechanism to encourage green innovation, so that rewards and punishments are clear, which can restrain enterprises to enhance the sense of social responsibility.

Although this paper tries its best to explore the impact of the enterprise digital transformation on green innovation from the theoretical and empirical perspectives, there are still several shortcomings: First, regarding the measurement of the enterprise digital transformation, the existing literature is mainly based on digital tangible assets, while this paper is based on the textual analysis method of machine learning, which not only includes digital tangible assets, but also contains more important digital intangible assets, such as senior digital transformation thinking and digital transformation strategy awareness. Of course, there are limitations, such as digital term dictionary relies too much on subjective judgment, and text analysis only focuses on the management discussion and analysis (MD&A) part, which may potentially affect the results of this paper. We will continue to pay attention to the latest research progress of digital transformation and continuously improve the indicator. Second, the discussion on the three mechanisms of digital transformation lacks rigorous mathematical proof, and we can consider unifying the three mechanisms into a theoretical model framework for analysis at a later stage, while the empirical study can consider using the three-step mediation effect method to measure the degree of influence of each mechanism, that is, the indirect effect. Third, the empirical process ignores the spillover effects of digital transformation and green innovation, which may overestimate the results of this paper as both are able to bring positive spillover effects to neighboring firms, so the introduction of spatial econometric models can be considered for examination in later studies. Fourth, the external validity of the findings has yet to be verified, and whether the findings of the study are equally applicable in other developing and developed countries requires empirical evidence. We will consider using data from other representative countries to test this later.

## Supporting information

S1 Data (ZIP)
